# Single-Step Incubation Determination of miRNAs in Cancer Cells Using an Amperometric Biosensor Based on Competitive Hybridization onto Magnetic Beads

**DOI:** 10.3390/s18030863

**Published:** 2018-03-15

**Authors:** Eva Vargas, Eloy Povedano, Víctor Ruiz-Valdepeñas Montiel, Rebeca M. Torrente-Rodríguez, Mohamed Zouari, Juan José Montoya, Noureddine Raouafi, Susana Campuzano, José M. Pingarrón

**Affiliations:** 1 Department of Analytical Chemistry, Faculty of Chemistry, University Complutense of Madrid, 28040 Madrid, Spain; evargas_orgaz@hotmail.com (E.V.); elpove01@ucm.es (E.P.); vrvmontiel@ucm.es (V.R.-V.M.); rebeca.magnolia@gmail.com (R.M.T.-R.); 2 Sensors and Biosensors Group, Laboratory of Analytical Chemistry and Electrochemistry (LR99ES15), Department of Chemistry, Faculty of Science, University of Tunis El Manar, Rue Béchir Salem Belkheria, Tunis El-Manar, 2092 Tunis, Tunisia; Mohamed.zouari@fst.utm.tn (M.Z.); noureddine.raouafi@fst.utm.tn (N.R.); 3 Cannan Research and Investment & Faculty of Medicine, University Complutense of Madrid, 28040 Madrid, Spain; jjmontoya@canaanrd.com

**Keywords:** miRNA, screen-printed electrode, amperometry, competitive assay, magnetic beads, cancer cells

## Abstract

This work reports an amperometric biosensor for the determination of miRNA-21, a relevant oncogene. The methodology involves a competitive DNA-target miRNA hybridization assay performed on the surface of magnetic microbeads (MBs) and amperometric transduction at screen-printed carbon electrodes (SPCEs). The target miRNA competes with a synthetic fluorescein isothiocyanate (FITC)-modified miRNA with an identical sequence for hybridization with a biotinylated and complementary DNA probe (b-Cp) immobilized on the surface of streptavidin-modified MBs (b-Cp-MBs). Upon labeling, the FITC-modified miRNA attached to the MBs with horseradish peroxidase (HRP)-conjugated anti-FITC Fab fragments and magnetic capturing of the MBs onto the working electrode surface of SPCEs. The cathodic current measured at −0.20 V (versus the Ag pseudo-reference electrode) was demonstrated to be inversely proportional to the concentration of the target miRNA. This convenient biosensing method provided a linear range between 0.7 and 10.0 nM and a limit of detection (LOD) of 0.2 nM (5 fmol in 25 μL of sample) for the synthetic target miRNA without any amplification step. An acceptable selectivity towards single-base mismatched oligonucleotides, a high storage stability of the b-Cp-MBs, and usefulness for the accurate determination of miRNA-21 in raw total RNA (RNA_t_) extracted from breast cancer cells (MCF-7) were demonstrated.

## 1. Introduction

Breast cancer (BC) is one of the three most common invasive cancers in females, with an estimated 1.5 million new cases per year [1], and one of the leading causes of cancer mortality among women worldwide [2]. Effective management of BC depends on early diagnosis and proper monitoring of the response of patients to therapy, which implies the need for identification of sensitive and specific biomarkers useful for early detection and disease monitoring [1].

Accumulated evidence in the past several years has highlighted the great promise of miRNAs as diagnostic, prognostic, or predictive biomarkers in the clinical management of patients with BC [3]. These miRNAs are short, non-coding single-stranded RNA (ssRNA) molecules, 19–23 nucleotides in length [4], acting at the post-transcriptional level by partial binding to the mRNA of genes [1,5,6]. However, the determination of miRNAs demands highly sensitive and selective methodologies due to their low abundance and high sequence similarity in family members. Moreover, their short length makes it difficult to use traditional DNA methods (primers and probe selection for amplification and sandwich assays) for their determination [7,8,9,10,11,12].

Current strategies for miRNA determination include miRNA microarrays [13,14,15,16], Northern blotting [17,18], and in situ hybridization and quantitative reverse-transcription polymerase chain reaction (qRT-PCR) [19,20,21]. However, the wide applicability of these strategies is hindered by their high cost, poor sensitivity, and the need for tedious analysis protocols. Furthermore, the requirement for skilled technicians and sophisticated and non-portable instruments restricts their use to central laboratories. Therefore, there is a pressing need to develop simple, sensitive, rapid, portable, and low-cost methodologies for the determination of miRNAs. Within this context, electrochemical biosensors have demonstrated very interesting features compatible with current clinic demands in terms of portability and simplicity for the determination of miRNAs.

On the other hand, it has been widely demonstrated that magnetic microbeads (MBs) offer very attractive characteristics in the development of electrochemical biosensors, such as lower limits of detection, faster assay kinetics, and smaller matrix effects [22,23,24,25]. These advantages have been coupled in many cases with those provided by planar disposable electrodes (versatility of fabrication and mass production at low cost) to develop different competitive electrochemical biosensing platforms for the quantification of miRNAs. Nevertheless, most of these platforms involve different preparation steps and require the use of expensive affinity reagents [26,27,28,29,30,31] and multiple probes in connection with amplification strategies [28,32].

With the aim of simplifying these strategies, we describe in this work the first competitive direct hybridization assay implemented onto the surface of MBs for the amperometric determination of miRNAs. Additional advantages of this methodology compared to our previous developments include simplicity, one-step incubation, lower cost, and not requiring the use of expensive affinity/amplification reagents and/or multiple probes. The method implies the competition of the target miRNA with a synthetic fluorescein isothiocyanate (FITC)-miRNA for the hybridization with a biotinylated DNA capture probe (b-Cp) immobilized onto Strep-MBs. As a model miRNA target, miRNA-21, a biomarker for many types of cancer and also cardiovascular disease [1,33], was chosen to exemplify the technique. Amperometric transduction was performed at screen-printed carbon electrodes (SPCEs) using the H_2_O_2_/Hydroquinone (HQ) system after labeling the FITC-miRNA immobilized onto the b-Cp-MBs with an anti-FITC-HRP. In this study, we show that this method allows the accurate determination of miRNA-21 in breast cancer cells without reverse transcription to cDNA, amplification, preconcentration, or purification steps.

## 2. Experimental

### 2.1. Apparatus and Electrodes

Amperometric measurements were made with a CH Instruments (Austin, TX, USA) 812B potentiostat controlled by CH Instruments software. Screen-printed carbon electrodes (SPCEs) (DRP-110, DropSens, Spain), consisting of a 4-mm diameter carbon working electrode, a carbon counter electrode, and an Ag pseudo-reference electrode, were used as electrochemical transducers in conjunction with a specific cable connector (DRP-CAC, DropSens, Llanera, Spain). All measurements were performed at room temperature. A neodymium magnet (AIMAN GZ, Madrid, Spain) embedded in a homemade Teflon casing was used to magnetically capture the modified-MBs on the surface of SPCEs.

A Raypa steam sterilizer, a biological safety cabinet Telstar Biostar, an incubator shaker Thermo-shaker MT100 (Universal Labortechnik, Leipzig, Germany), a Bunsen AGT-9 vortex for homogenization of the solutions, a magnetic particle concentrator DynaMag™-2 (123.21D, Invitrogen Dynal AS, Oslo, Norway), and a Sigma 1–15 K refrigerated microcentrifuge were also employed. The quality and quantity of the extracted cellular RNA were evaluated by using a NanoDrop^®^ ND-1000 spectrophotometer (NanoDrop Technologies, Wilmington, DE, USA).

### 2.2. Reagents and Solutions

All reagents were of the highest analytical grade. Streptavidin-modified magnetic beads (Strep-MBs, 2.8 µm Ø, 10 mg·mL^−1^, Dynabeads M-280 Streptavidin, 11206D) were purchased from Dynal Biotech ASA.

NaCl, KCl, NaH_2_PO_4_, Na_2_HPO_4_, and Tris–HCl were purchased from Scharlab. Hydroquinone (HQ) and H_2_O_2_ (30%, *w*/*v*) were purchased from Sigma-Aldrich and ethylenediaminetetraacetic acid (EDTA) from Merck (Darmstadt, Germany). Anti-fluorescein (FITC) Fab Fragments conjugated with HRP (anti-FITC-HRP) were purchased from Roche. A blocker casein solution (BB solution, a ready-to-use, PBS solution of 1% *w*/*v* purified casein) was purchased from Thermo Scientific. All the DNA and RNA synthetic oligonucleotides (sequence described in Table 1, purchased from Sigma-Aldrich, St. Louis, MO, USA) were reconstituted upon reception in nuclease-free water to a final concentration of 100 μM, divided into small aliquots, and stored at −80 °C.

All the required buffer solutions were prepared in deionized water from a Millipore Milli-Q purification system (18.2 MΩ cm): phosphate-buffered saline (PBS) consisting of 0.01 M phosphate buffer solution containing 0.137 M NaCl and 0.0027 M KCl, pH 7.5; Binding and Washing buffer (B&W) consisting of 10 mM Tris–HCl solution containing 1 mM EDTA and 2 M NaCl, pH 7.5 (sterilized after their preparation by autoclaving at 120 °C for 15 min); PBS:BB (1:1) solution prepared by mixing equal volumes of PBS and BB solutions and 0.05 M phosphate buffer of pH 6.0.

### 2.3. MBs Modification

A 0.5-μL aliquot of the commercial Strep-MBs suspension was transferred into a microcentrifuge tube and washed twice with 50 μL of B&W buffer. Between washes, the particles were placed in the magnetic concentrator and, after 3 min, the supernatant was discarded. Washed MBs were incubated for 15 min at 30 °C under continuous stirring (950 rpm) with 25 μL of 2.5 nM of the b-Cp solution (prepared in B&W). After two washing steps with 50 μL of a PBS:BB (1:1) solution, the b-Cp-MBs can be stored (in 50 μL filtered PBS at 4 °C) or used directly to perform the determination. In this latter case, the b-Cp-MBs were incubated for 120 min (950 rpm, 30 °C) in 25 μL of a mixture solution containing the synthetic target miRNA (or 0.5–2.0 μg of the extracted RNA_t_), 2.5 nM FITC-miRNA, and 1/500 diluted anti-FITC-HRP (prepared also in PBS:BB (1:1)). After washing twice with 50 μL of PBS:BB (1:1) solution, the modified-MBs were re-suspended in 50 μL of 0.05 M sodium phosphate buffer solution (pH 6.0) to perform the amperometric detection.

All the MB manipulations carried out before the amperometric measurements were made in a laminar flow cabinet to avoid RNAse contamination and prevent miRNA degradation. Moreover, control experiments in the absence of target miRNA were performed daily to evaluate the blank signal.

### 2.4. Electrochemical Measurements

Amperometric measurements were made by pipetting the 50 μL of the modified MBs suspension onto the SPCE after placing it on a homemade casing of Teflon with an encapsulated neodymium magnet. In this way, the MBs were magnetically captured on the working carbon electrode in a reproducible and stable manner. Then, the SPCE/magnet holding block ensemble was immersed into an electrochemical cell containing 10 mL of 0.05 M phosphate buffer of pH 6.0 supplemented with 1.0 mM HQ (prepared just before performing the electrochemical measurement). The amperometric measurement was made at −0.20 V versus an Ag pseudo-reference electrode in stirred solutions. Once the background current was stabilized, 50 μL of a freshly prepared 0.1 M H_2_O_2_ solution were added and the cathodic current recorded until the steady-state current was reached (~100 s). All of the amperometric signals represent the difference between the steady-state and the background currents. Data shown correspond to the average of at least three replicates with the confidence intervals calculated for α = 0.05. The limits of detection (LOD) and quantification (LOQ) were estimated according to the 3 × s_b_/m and 10 × s_b_/m criteria, respectively, with s_b_ being the standard deviation for 10 measurements made with no target miRNA and m the slope value of the calibration plot obtained with the synthetic target miRNA.

### 2.5. Cultured Cells and RNA_t_ Extraction

Protocols used for cultured cells and RNA_t_ extraction were the same as described previously [26].

Briefly, non-tumorigenic epithelial MCF-10A cells were grown at 37 °C in a humidified atmosphere containing 5% CO_2_ in high-glucose DMEM/Ham’s Nutrient Mixture F12 (1:1) with 2.5 mM l-glutamine (GIBCO-Invitrogen, Carlsbad, CA, USA), 5% horse serum (Gibco), 10 mg mL^−1^ human insulin (Sigma, St. Louis, MO, USA), 0.5 mg mL^−1^ hydrocortisone (Sigma), 10 ng mL^−1^ epidermal growth factor (EGF), and 100 ng mL^−1^ cholera toxin (QuadraTech Ltd., Epsom, UK). Breast cancer cells MCF-7 were cultured in high-glucose DMEM (Dulbecco’s modified Eagle’s medium) supplemented with 10% fetal bovine serum, 100 U mL^−1^ penicillin, 100 μg mL^−1^ streptomycin, and 2.5 mM l-glutamine.

For RNA_t_ isolation, cells were washed with PBS, scraped off, and spun down. The pellet was homogenized in Tri Reagent (Molecular Research Center, Inc., Cincinnati, OH, USA) for 5 min at room temperature before chloroform extraction and the RNA_t_, in the upper aqueous phase, was precipitated with isopropylalcohol and washed twice in 70% EtOH. The pellet was dried out in a heating plate for 10 min at 80 °C and, then, dissolved in RNase-free water, and stored at −80 °C [26]. The absorbance ratio values measured at the appropriate wavelengths (260, 230, and 280 nm) with an ND-1000 spectrophotometer confirmed in all cases pure RNA.

## 3. Results and Discussion

**Figure 1:**
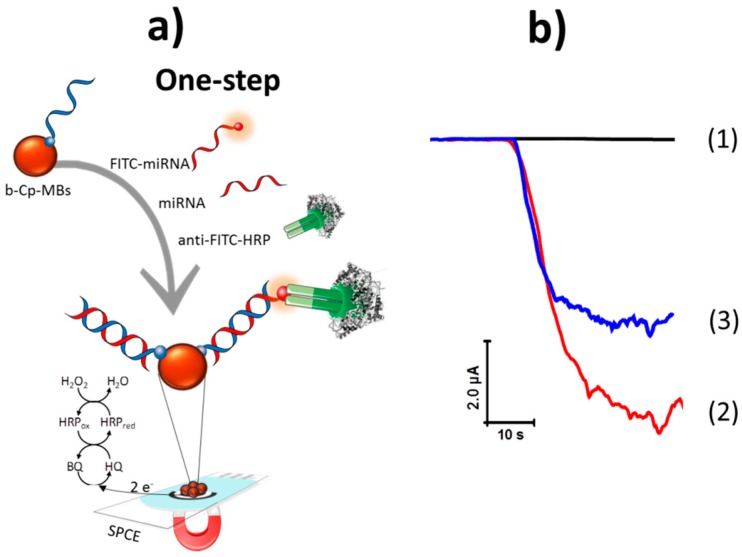
(**a**) Schematic display of the amperometric biosensor developed for miRNA-21 determination based on a competitive DNA/RNA hybridization assay onto magnetic beads (MBs) and amperometric detection using the H_2_O_2_/ Hydroquinone (HQ) system at a screen-printed carbon electrode (SPCE). (**b**) Amperometric responses obtained with the developed biosensor in the absence (1) and in the presence of 2.5 nM FITC-miRNA (2) and 5.0 nM of miRNA-21 (3). HRP: horseradish peroxidase, BQ: 1,4-hydroquinone.

The method reported here relies on a competitive DNA/RNA hybridization using a biotinylated DNA capture probe (b-Cp), complementary to the target miRNA, immobilized onto Strep-MBs and a fluorescein isothiocyanate (FITC)-modified synthetic RNA (FITC-miRNA) whose sequence is identical to that of the target miRNA. The b-Cp-modified MBs were incubated in the sample solution supplemented with fixed concentrations of the FITC-miRNA and anti-FITC-HRP so that the FITC-miRNA competed with the target miRNA for hybridization with the b-Cp immobilized onto the Strep-MBs. Therefore, the higher the concentration of the target miRNA, the lower the amperometric response measured using H_2_O_2_ as enzyme substrate and HQ as redox mediator due to the lower number of FITC-miRNA molecules and therefore of anti-FITC-HRP attached to the MBs (see Figure 1a).

Figure 1b, amperogram 1, shows that no significant amperometric response was recorded in the absence of FITC-miRNA because there was no attached anti-FITC-HRP. As expected, the largest amperometric signal was obtained when the electrode was incubated in a solution containing only the synthetic FITC-miRNA (amperogram 2 in Figure 1b) in agreement with the larger number of attached anti-FITC-HRP. However, this response decreased notably in the presence of both the FITC-miRNA and the target miRNA (amperogram 3 in Figure 1b) due to their competitive hybridization with the b-Cp, leading to a lower amount of FITC-miRNA molecules and therefore of anti-FITC-HRP attached on the MBs. These results demonstrated the feasibility of the direct competitive hybridization assay onto b-Cp-MBs for the quantification of miRNAs.

All of the experimental variables involved in the preparation of the competitive hybridization-based biosensor for miRNA determination were optimized. The adopted selection criterion was the largest current ratio between the values measured at a previously optimized detection potential for the HRP/HQ/H_2_O_2_ system of −0.20 V (versus the Ag pseudo-reference electrode), [34], in the absence (S_0_) and in the presence of 5.0 nM (S_1_) synthetic miRNA-21 (S_0_/S_1_ ratio). Table 2 summarizes the tested variables, their checked ranges, and the values selected for further work.

**Figure 2:**
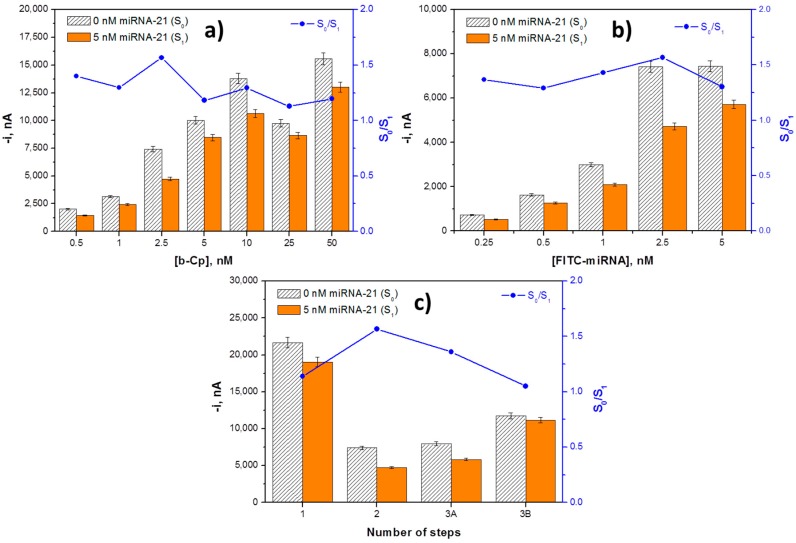
Dependence of the amperometric signals measured with the competitive DNA/RNA hybridization biosensor in the absence (S_0_) and in the presence of 5.0 nM miRNA-21 (S_1_), and the corresponding S_0_/S_1_ ratio with the b-Cp (**a**) and FITC-miRNA (**b**) loadings and with the number of steps involved in the biosensor preparation (**c**). Error bars estimated as three times the standard deviation of three replicates.

Two key variables in competitive hybridization assays are the loadings of the capture probe (b-Cp) and tracer agent (FITC-miRNA), which should be carefully optimized and kept fixed in the assays [35]. The b-Cp loading was tested between 0.5 and 50.0 nM. As it can be observed in Figure 2a, the largest S_0_/S_1_ ratio was found for 2.5 nM. For lower loadings, the responses in the absence of target miRNA were very low as a result of the small number of immobilized FITC-miRNA molecules. Conversely, larger loadings provoked a decrease in the S_0_/S_1_ ratio due to a hindered competition in the presence of large probe concentrations. This optimal low probe concentration is in agreement with the behavior expected in competitive configurations where higher sensitivity is obtained for a lower probe concentration, unlike what happens in sandwich formats [35]. The dependence of the amperometric signals with the FITC-miRNA concentration was tested over the 0.25 to 5 nM range. Data displayed in Figure 2b show that a larger S_0_/S_1_ ratio was found using a 2.5 nM concentration of FITC-miRNA, where a sufficiently large amperometric response was obtained in the presence of the target analyte.

Moreover, the sequence of the different steps involved in the biosensor preparation protocol may have an important effect on the assay performance. The different protocols evaluated, all involving 30 min incubation steps, include:

- Protocol 1, single step: incubating the Strep-MBs with a mixture solution containing b-Cp, target miRNA-21, FITC-miRNA, and anti-FITC-HRP.
- Protocol 2, 2 steps: (1) immobilization of the b-Cp onto the Strep-MBs and (2) incubation of the b-Cp-MBs with a mixture solution containing target miRNA-21, FITC-miRNA, and anti-FITC-HRP.
- Protocol 3A, 3 steps: (1) immobilization of the b-Cp onto the Strep-MBs, (2) hybridization of the miRNA-21 onto the b-Cp-MBs, and (3) incubation of the miRNA-21/b-Cp-MBs with a mixture solution containing FITC-miRNA and anti-FITC-HRP.
- Protocol 3B, 3 steps: (1) immobilization of the b-Cp onto the Strep-MBs, (2) incubation of the b-Cp-MBs with a mixture solution containing target miRNA-21 and FITC-miRNA, and (3) labeling of the FITC-miRNA attached to the b-Cp-MBs with the anti-FITC-HRP.

The amperometric responses measured for 0.0 and 5.0 nM of target miRNA-21 as well as the corresponding S_0_/S_1_ ratios using the different protocols are compared in Figure 2c. As can be observed, although protocols 1, 2, and 3A allowed for discrimination between the absence and presence of the target miRNA, the largest S_0_/S_1_ ratio was achieved following protocol 2, which was selected to implement the biosensing strategy. The poor discrimination observed using protocol 3 can be attributed to a less-favourable competition with FITC-miRNA labeled with the anti-FITC-HRP. The lack of discrimination observed using protocol 3B could be attributed to a hindered recognition of the FITC-miRNA, once immobilized on MBs, by the anti-FITC-HRP.

### 3.1. Analytical Characteristics

**Figure 3:**
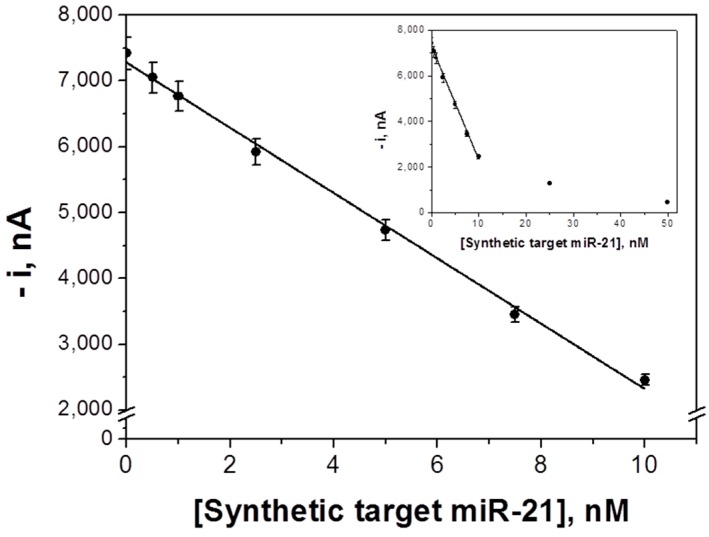
Calibration plot constructed for synthetic target miRNA-21 with the competitive DNA/RNA hybridization biosensor. Inset shows the saturation effect at higher concentrations of the target miRNA. Error bars estimated as three times the standard deviation of three replicates.

The calibration curve constructed for the amperometric determination of the synthetic target miRNA is displayed in Figure 3. As expected for a competitive hybridization assay, the larger the concentration of target miRNA the lower the amperometric response as a consequence of the lower concentration of FITC-miRNA and consequently of anti-FITC-HRP attached to the MBs. A linear dependence (*r* = 0.999) of the current values measured with the miRNA-21 concentration was found from 0.7 to 10.0 nM, with slope and intercept values of (−496 ± 12) nA nM^−1^ and (7279 ± 65) nA, respectively. The calculated LOD and LOQ values were 0.2 and 0.7 nM (5 and 17.5 fmol in 25 μL of sample), respectively.

The achieved LOD is similar (0.2 versus 0.4 nM) [27] or higher than those reported for other MBs-based approaches for the determination of miRNAs (6 aM [28], 0.4 pM [31], 2.4 pM [29], 40 pM [26], and 60 pM [32]). However, the methodology reported here is carried out in a single incubation step (once the MBs were modified with the b-Cp) with a very low concentration of synthetic nucleic acid probe (2.5 nM) and without any amplification strategy. In addition, no expensive affinity bioreceptors [26,27,28,29,30,31] were needed. These features greatly simplify the potential implementation of this technology in portable and automatic devices suitable to carry out the determination at affordable cost in different settings. It is also worthwhile mentioning that the sensitivity achieved is sufficient for relevant applications as are shown in Section 3.3. Moreover, it is worth mentioning that although 2 h is the optimal time, the determination could be performed in half the time (1 h) losing 66% of the sensitivity.

The amperometric responses for 5.0 nM miRNA-21 provided by 10 different biosensors prepared in the same manner exhibited a high reproducibility with a relative standard deviation (RSD) value of 3.3%, thus confirming the suitability of both the sensor fabrication and the amperometric transduction protocols used.

The storage stability of the b-Cp-MBs was evaluated by keeping them at 4 °C in microcentrifuge tubes containing 50 μL of filtered PBS. Each working day, the amperometric responses obtained with the biosensors prepared using the stored b-Cp-MBs for 0.0 and 5.0 nM synthetic miRNA-21 solutions were compared. Interestingly, no significant decrease in the resulting S_0_/S_1_ ratio (not shown) was observed during 49 days (no longer times were evaluated). This behavior creates the possibility of preparing and then storing the b-Cp-MBs for at least this amount of time until biosensor preparation is required.

### 3.2. Selectivity

**Figure 4:**
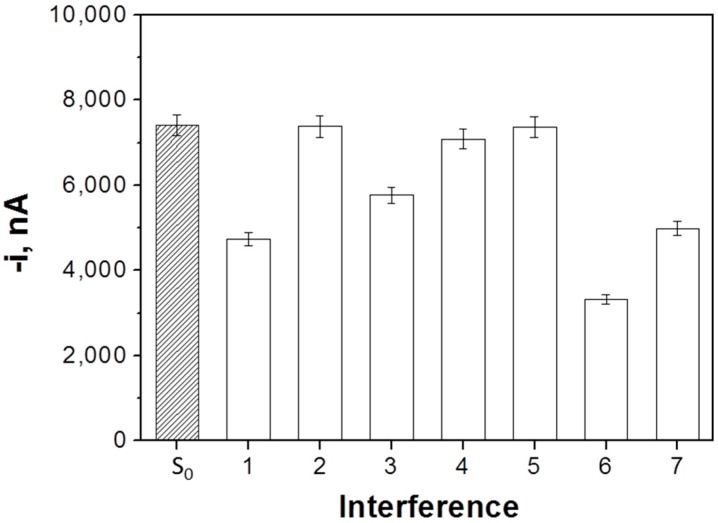
Selectivity of the developed competitive DNA/RNA hybridization-based biosensor for determination of miRNA-21. Amperometric responses measured in the absence (S_0_) and in the presence of 5.0 nM miRNA-21 (1), 1-m (c) (2), 1-m (t) (3), NC1 (miRNA-205) (4), NC2 (miRNA-122) (5), and mixture solutions containing the target miRNA and the two 1-m (6) or the target miRNA and the two non-complementary (NC) sequences (7). Error bars estimated as three times the standard deviation of three replicates.

The selectivity of the developed biosensor was checked by comparing the amperometric signals measured in the absence (S_0_) and in the presence of 5.0 nM miRNA-21 with those recorded in the presence of two different single-base mismatched in central (1-m(c)) or terminal (1-m(t)) position sequences, two fully non-complementary (NC) sequences (corresponding to miRNA-205 and miRNA-122), and mixtures containing the target miRNA and the two 1-m (mixture 1) or the two NC (mixture 2) sequences (each at a 5.0 nM concentration). As it is shown in Figure 4, the amperometric responses obtained with the NC sequences and the 1-m (c) were similar to those measured in the absence of target miRNA, thus proving that these sequences cannot compete with the FITC-miRNA for the b-Cp immobilized onto the MBs. Moreover, the amperometric response obtained for 1-m (t) is intermediate between the responses obtained in the absence and in the presence of target RNA, which is in agreement with a less-favorable hybridization with this sequence due to the presence of the mismatched base in the terminal position of the hybrid. The amperometric signals obtained for the mixture solutions are in agreement with the results for the individual sequences. The responses recorded for the mixtures prepared in the presence of the 1-m and the NC sequences are lower and similar to that obtained in the presence of the target miRNA alone. These results confirm the high selectivity of the developed biosensor against the NC and 1-m (c) sequences. The selectivity towards 1-m (t) can be considered acceptable. Considering the low probability that the target miRNA was in the sample with 1-m sequences at the same concentration level and with the mismatched base in terminal position [36], this fact cannot be considered as an important problem for the applicability of the biosensor. Indeed, the results achieved with the two 1-m sequences demonstrated the potential ability of the developed biosensor to detect the existence and even the position of a single mismatched base.

### 3.3. Determination of Mature miRNA-21 in RNA_t_ Extracted from Cancer Cells

The clear association between the hyperexpression of miRNA-21 and the incidence and progression of breast cancer [37,38,39,40] led us to evaluate the applicability of the developed biosensor by determining the mature content of the target miRNA in breast cancer (MCF-7) and non-tumoral breast primary epithelial (MCF-10A) cells.

**Figure 5:**
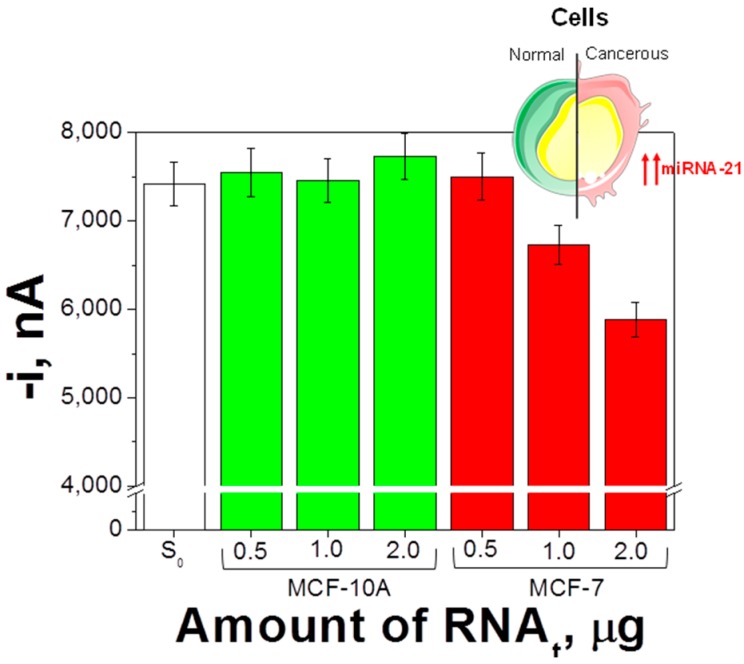
Determination of the endogenous content of miRNA-21 in raw total RNA (RNA_t_) extracted from breast cells. Amperometric responses measured with the developed biosensor in the absence (S_0_) and in the presence of different amounts of RNA_t_ extracted from MCF-10A and MCF-7 cells. Error bars estimated as three times the standard deviation of three replicates.

The amperometric signals provided by the biosensor in the analysis of different amounts of RNA_t_ extracted from both types of cell are shown in Figure 5. As expected, substantially lower amperometric responses were measured for RNA_t_ extracted from the MCF-7 when compared with those from MCF-10A cells according to the overexpression of the oncogenic target miRNA in the cancerous cells [7,26,27,32,41] as long as the amount of extracted RNA_t_ from MCF-7 cells was at least 1 μg.

Moreover, the slope value of the linear calibration plot constructed for miRNA-21 in the presence of 1.0 μg of RNA_t_ extracted from MCF-10A (−564 ± 87) nA·nM^−1^ was not significantly different than that measured for miRNA-21 in buffered solutions (−496 ± 12) nA·nM^−1^. Therefore, no apparent matrix effect occurred using this RNA_t_ amount and the endogenous content of the target miRNA in the MCF-7 cells could be estimated by simple interpolation of the amperometric responses measured for these cells into the calibration graph constructed with miRNA-21 standards (Figure 4). Results obtained were (25 ± 4) amol miRNA-21 ng^−1^ RNA_t_ (RSD*_n_*_=5_ = 12.1%), which were similar to those reported by other authors, (18 ± 2) [26], 21.7 [27], and (21 ± 3) amol miRNA-21 ng^−1^ of RNA_t_ [32], using other electrochemical biosensing strategies. It is also useful to note that the reported levels of miRNA-21 in MCF-10A cells are on the order of 1.0 amol ng^−1^ RNA_t_, while they are in the range of 0.1−1.5 and 0.4−3.9 amol per ng RNA_t_ in breast normal and tumor tissues, respectively [29,42].

Regarding the comparison with other electrochemical methods applied to the determination of the endogenous content of a target miRNA in cell lysates [26,27,29,30,31,32], as well as with other types of detection [43], the most relevant advantage of the methodology reported here is the simplicity of the determination in one single step, without long and expensive chemistry to label the target miRNA, and using just 1.0 μg of the extracted RNA_t_.

These results demonstrated an acceptable reproducibility of the whole method, including the RNA_t_-extraction protocol and the determination with the developed electrochemical biosensor, as well as the feasibility and potential applicability of the developed approach to perform a simple and accurate determination of the target miRNA in just one incubation step and directly in raw RNA_t_ extracted from cancerous cells, without reverse transcription, amplification, preconcentration, or labeling steps. Moreover, the straightforward determination in this raw RNA_t_ matrix, where many other non-target miRNAs are also present to a large extent, further highlights the excellent selectivity of the developed methodology.

## 4. Conclusions

This work describes an amperometric biosensor based on a competitive DNA/RNA hybridization format implemented on the surface of MBs and SPCEs for the determination of miRNA-21. This strategy exhibits an attractive analytical performance with a LOD of 0.2 nM (5.0 fmol in 25 μL of sample), a good reproducibility between the amperometric responses provided by different biosensors prepared in the same manner, a clear and acceptable discrimination towards non-complementary and 1-m sequences, respectively, and a 49-day storage stability of the magnetic bioconjugates.

It is worth mentioning also that the developed methodology allowed for the reliable and accurate determination of the target miRNA in cancer cells involving a simple and 1-step protocol (once the b-Cp-MBs were prepared) without requiring previous reverse transcription to cDNA, amplification, preconcentration, or purification steps. This performance makes it ideal for routine determinations in both clinical and research settings and as a basis for future diagnostic point-of-care (POC) devices. Moreover, although focused only on the determination of miRNA-21 as model, the same methodology can be applied to the determination of any miRNA in single or multiplexed formats simply by modifying the Strep-MBs with the appropriate complementary biotinylated DNA capture probes.

## Figures and Tables

**Table 1:** Oligonucleotides used in this work.

Oligonucleotide	Sequence (5′→3′)
Biotinylated capture probe (b-Cp)	TCAACATCAGTCTGATAAGCTA-Biotin
Target miRNA-21	UAGCUUAUCAGACUGAUGUUGA
FITC-modified miRNA (FITC-miRNA)	UAGCUUAUCAGACUGAUGUUGA-FITC
1-mismatched in central position (1-m(c))	UAGCUUAUCAAACUGAUGUUGA
1-mismatched in terminal position (1-m(t))	UAGCUUAUCAGACUGAUGUUGG
Non-complementary 1 (NC1, miRNA-205)	UCCUUCAUUCCACCGGAGUCU
Non-complementary 2 (NC2, miRNA-122)	UGGAGUGUGACAAUGGUGUUUG

FITC: fluorescein isothiocyanate.

**Table 2:** Optimization of the experimental variables affecting the performance of the amperometric biosensor developed for miRNA-21 determination.

Experimental Variable	Tested Range	Selected Value
Strep-MBs, μL	0.25–5.0	0.5
(b-Cp), nM	0.5–50.0	2.5
Incubation time with b-Cp, min	15–60	15
(FITC-miRNA), nM	0.25–5.0	2.5
anti-FITC-HRP	1/5000–1/250	1/500
Number of steps	1–4	2
Incubation time with mixture (miRNA-21 + FITC-miRNA + anti-FITC-HRP), min	15–270	120
